# Correction: The splicing regulator PTBP2 controls a program of embryonic splicing required for neuronal maturation

**DOI:** 10.7554/eLife.31550

**Published:** 2017-10-09

**Authors:** Qin Li, Sika Zheng, Areum Han, Chia-Ho Lin, Peter Stoilov, Xiang-Dong Fu, Douglas L Black

Li Q, Zheng S, Han A, Lin C-H, Stoilov P, Fu X-D, Black DL. 2014. The splicing regulator PTBP2 controls a program of embryonic splicing required for neuronal maturation. *eLife* **3**:e01201. doi: 10.7554/eLife.01201.

Published 21, January 2014

It has come to our attention that there is an error in Figure 1D of our paper. The size of the band indicated as the knockout allele is incorrect for the primers reported. We also found that we failed to provide a protocol for the genotyping primers reported in the paper. In subsequent work, we have developed additional primers for genotyping this mouse that work more efficiently and have sequenced the floxed and deleted alleles to confirm their structure. We present here corrected versions of Figure 1B and 1D showing the positions of the new primers, their expected product sizes, and a gel of these PCR products from various genotypes. The sequences of these primers and the PCR protocol for their use as well as that for the original primers are now described in the Materials and Methods of the main article. The genomic DNA sequence of PTBP2^loxP^ is reported in Supplementary file 9. While this error does not affect the rest of the paper and its conclusions, we regret any confusion or difficulties in genotyping it may have caused.

The corrected Figure 1 is shown here:

**Figure 1:**
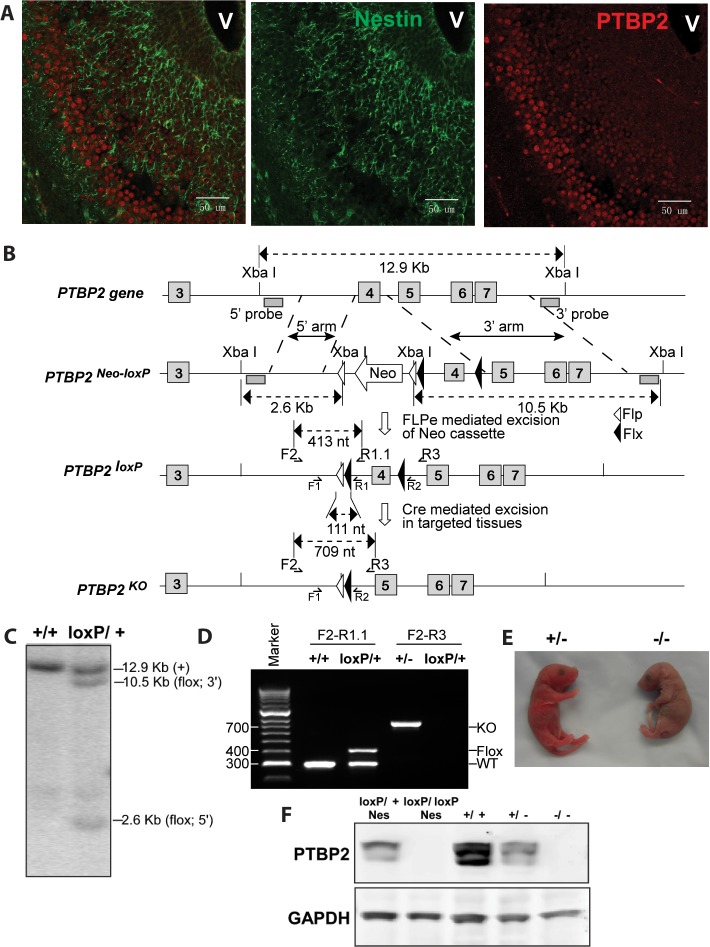


The originally published Figure 1 is shown for reference:

**Figure 2:**
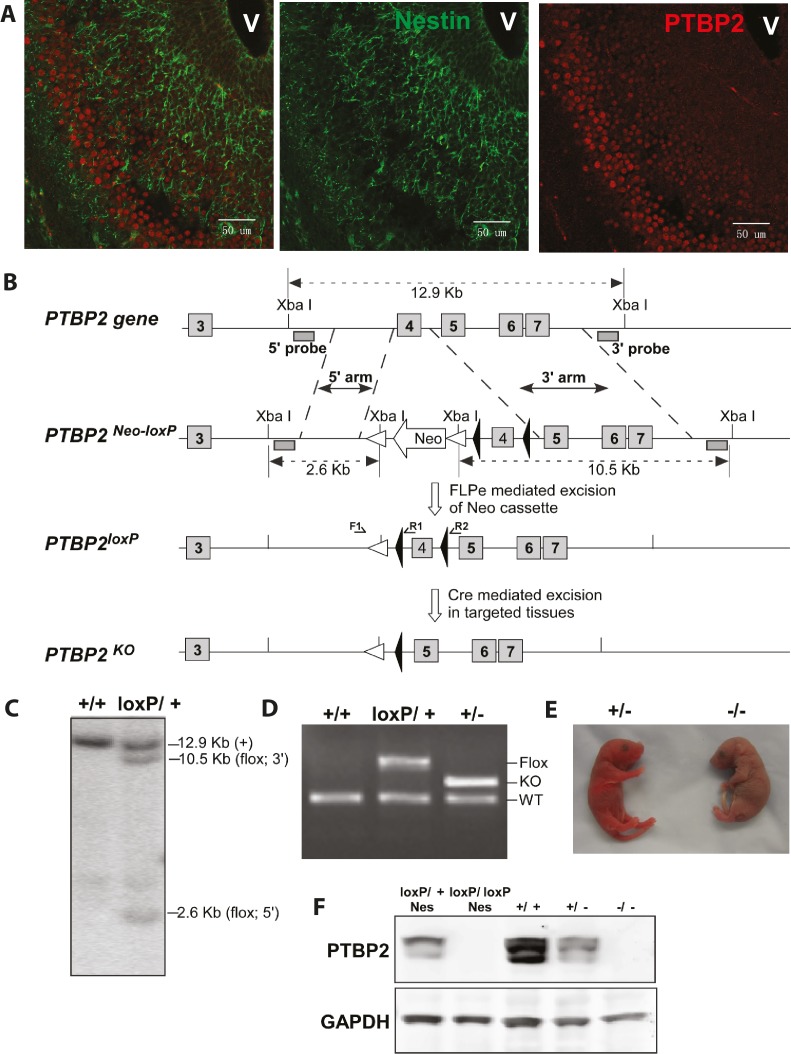


CORRECTED:

Genotyping PCR primers were TCTACTTCATTGTGTTGTTTTG (F1), AGGCATCCATAATTACACAGTGT (F2), GATACAGCAGGCTCCCCTCA (R1), AAGTGATACAGCAGGCTCCC (R1.1), ATAAGCATTTTCTAGCACCAA (R2) and GCGCGTATCCTCAAACAAGAC (R3). 50-100 ng of purified genomic DNA was amplified by PCR using 5 μL of 2x GoTaq Green Master Mix (Promega™ M7123) with 0.5 uM of each PCR primer in a total 10 μL reaction. The F2 and R1.1 pair yields a 302 bp product for the WT and a 413 bp product for the loxP allele. Initial denaturation was at 95°C for 2 min, followed by 30 cycles of denaturation at 95°C for 30 sec, annealing at 53°C for 30 sec and extension at 72°C for 40 sec, and final extension at 72°C for 6 min. The F2 and R3 pair yields a 709 bp product for the loxP deleted allele (KO). For these primers, initial denaturation was at 95°C for 2 min, followed by 31 cycles of denaturation at 95°C for 30 sec, annealing at 60°C for 30 sec and extension at 72°C for 40 sec, and final extension at 72°C for 6 min. The F1 and R1 primer pair yields a 184 bp product for the WT allele and 295 bp product for the loxP allele. The F1 and R2 pair yields a 455 bp product for the loxP deleted allele (KO). For these primer pairs, initial denaturation was at 94°C for 4 min, followed by 35 cycles of denaturation at 94°C for 30 sec, annealing at 55°C for 30 sec and extension at 72°C for 30 sec, and final extension at 72°C for 5 min.

ORIGINAL:

Genotyping PCR primers were TCTACTTCATTGTGTTGTTTTG (F1) and GATACAGCAGGCTCCCCTCA (R1), ATAAGCATTTTCTAGCACCAA (R2).

The article has been corrected accordingly.

