# Supplementary material for: The splicing regulator PTBP2 controls a program of embryonic splicing required for neuronal maturation
Source: eLife. 2014 Jan 21;3:e01201. doi: 10.7554/eLife.01201 (PMC3896118; doi:10.7554/eLife.01201)
Supplement: Supplementary file 9. — DOI: http://dx.doi.org/10.7554/eLife.01201.015 [file elife-01201-supp9.docx]

**Genomic DNA Sequence (PTBP2^loxP^)**

**>F2**

|

GGCTTTCAGTTATGTATGAAATAATCTAT**AGGCATCCATAATTACACAGT** < 50

**GT**TTTTCTTTTTGATGGAACTTTGTAATGAAGGCATATTTATTTATTGAA < 100

ATGAACACTACTGGTCTAATGTAATGATCCAAAAGTATATACT**TCTACTT** < 150

**>F1**

|

**CATTGTGTTGTTTTG**TTTTATTATTTATTCTTAGTAAGGTCCTGTGTC**ga** < 200

**cggtatcgataagcttgatggGAAGTTCCTATTCTCTAGATAGTATAGGA** < 250

**>1stFloxinsertion**

|

**ACTTCgatATAACTTCGTATAATGTATGCTATACGAAGTTATgatgggga** < 300

**tcctgtgtc**CTGTTTAAGCCTTACCATATGCATGTAACATATAGAAAAAT < 350

CTTCTATAGAGTTTGATTCTAGCTGTTTATCCATGTGAGTGGGGGCTTTG < 400

**>R1.1**

**>R1**

| |

GGATATATCTCTTTTGGC**TGAGGGGAGCCTGCTGTATCACTT**AACTTGTA < 450

CAAAGAATTCAATGGTAATTTTAATTTACTTGTGTTCTTTAGTCATTGGA < 500

CAGTTTTGGTATGAAGTTGAATTTTAGTATTTTTGCTAATTATTATTCTA < 550

CATAGACTGCACATGGCTTGATGGTAACCTTAGTTGATGGTTTAATGATT < 600

TTTCTCCTTTCCATTGAAAGCCTAATTTTAGCTTTTCAAGCATTCAGATT < 650

TACTCACCTATGTTTTAAAGTATTTTTAACTTTTCATAGGAATCCATTTT < 700

TTTCATACTGATTATAAAGAATATTTCTGTATGTCTTTCAG**CCAACGGTA** < 750

**ACGATAGTAAGAAATTTAAAGGAGAAGATAAAATGGATGGGGCTCCCTCT** < 800

**>Exon4**

|

**CGTGTACTTCACATTCGAAAGTTACCTGGTGAAGTGACTGAAACAGAAGT** < 850

**TATTGCTTTAGGTTTACCTTTTGGTAAGGTGACTAACATCCTTATGCTGA** < 900

**AAGGAAAAAACCAG**GTACAGTGCACAAGCGTTACTACTAGCATGTTAAAT < 950

TACACAGAAGGAGGCTTATCTGTCACCTAAAGGATTCATTGTCTGACAAA < 1000

ATTAAAAGAAGTTCAGTTTCCTGTTTGTTTTGTTTTGTAATAGCAGAAAC < 1050

CAGGGGTACTAGTAGAATCTATTACATGAGTTTATTCATCTGGTAGCATT < 1100

AGAAATCAGGCTTAAATTTTGGAT**ccggaacccttaatATAACTTCGTAT** < 1150

**>2ndfloxinsertion**

|

**AATGTATGCTATACGAAGTTATtaggtccctcgacctgcagcccaagctc** < 1200

**tcgagtttattcatctggtagcattagaaatcaggcttaaattttggat**G < 1250

CTCATTCATTAACAGTAGCAGTAGGACCTATTCATATTAAAAATGACATT < 1300

ATGTTTTAAAATTTCCAAAAATTTTATTTAGAAGAATTTATTTAATATTA < 1350

GAAAATTAATATTCTCTAAACGTAGCTAATATAATAATTATTCCGTCCTT < 1400

TTAATTTTTCAGTTGTATTTTTTATATGAGACCTTTAGGATAGTAACATA < 1450

**>R2**

|

AAATT**TTGGTGCTAGAAAATGCTTAT**TTTCTCTAGTTTTTTTAAAAAACA < 1500

GATTTATGGGATGTAGTAAGTTGTAGATGGAACCAACAAAATCGAAGGGT < 1550

ATTACTTGAATCAACCCCAATATTATTTTAAAAATTAAGTGATTTCTAAG < 1600

**>R3**

|

ACATGTTTTATTATTTTCAAATGATAT**GTCTTGTTTGAGGATACGCGC**AT < 1650

GTGAGCTAAGGCCCCAGCAGAAGCCTGTGGGCGTCCATTCCTTGGGATTG < 1700

GAGTTCCAGGCAAGGCTTCTGTAAGCCTTGTGCATGGTGA < 1740

Features :

F2 : [30 : 52]

R1 : [419 : 438]

R1.1 : [423 : 442]

R2 : [1456 : 1476]

R3 : [1628 : 1648]

Exon4 : [742 : 914]

1stFloxinsertion : [199 : 309]

2ndfloxinsertion : [1125 : 1249]

F1 : [144 : 165]
